# The cirrhosis care Alberta (CCAB) protocol: implementing an evidence-based best practice order set for the management of liver cirrhosis - a hybrid type I effectiveness-implementation trial

**DOI:** 10.1186/s12913-020-05427-8

**Published:** 2020-06-18

**Authors:** Michelle Carbonneau, Ejemai Amaize Eboreime, Ashley Hyde, Denise Campbell-Scherer, Peter Faris, Leah Gramlich, Ross T. Tsuyuki, Stephen E. Congly, Abdel Aziz Shaheen, Matthew Sadler, Marilyn Zeman, Jude Spiers, Juan G. Abraldes, Benjamin Sugars, Winnie Sia, Lee Green, Dalia Abdellatif, Jeffrey P. Schaefer, Vijeyakumar Selvarajah, Kaleb Marr, David Ryan, Yolande Westra, Neeja Bakshi, Jayant C. Varghese, Puneeta Tandon

**Affiliations:** 1 Alberta Health Services, Edmonton & Calgary, AB Canada; 2 Department of Medicine, Division of Gastroenterology, University of Alberta, Edmonton, AB T6G2X8 Canada; 3 Department of Family Medicine, University of Alberta, Edmonton, AB Canada; 4 Office of Lifelong Learning and the Physician Learning Program, Faculty of Medicine and Dentistry, University of Alberta, Edmonton, AB Canada; 5 Department of Medicine, Division of Cardiology, University of Alberta, Edmonton, AB Canada; 6 Department of Pharmacology, University of Alberta, Edmonton, AB Canada; 7 Department of Medicine, Division of Gastroenterology and Hepatology, University of Calgary, Calgary, AB Canada; 8 O’Brien Institute of Public Health, University of Calgary, Calgary, AB Canada; 9 Faculty of Nursing, University of Alberta, Edmonton, AB Canada; 10 Centre of Excellence for Gastrointestinal Inflammation and Immunity Research (CEGIIR), University of Alberta, Edmonton, AB T6G2X8 Canada; 11 Department of Medicine, Division of General Internal Medicine, University of Alberta, Edmonton, AB Canada; 12 Department of Medicine, Division of General Internal Medicine, University of Calgary, Calgary, AB Canada; 13 Central Alberta Digestive Disease Specialists, Red Deer, AB Canada

**Keywords:** Cirrhosis, Order set, Normalization process theory (NPT), Reach-effectiveness-adoption-implementation-maintenance (RE-AIM), Hybrid trial, Consolidated framework on implementation research (CFIR)

## Abstract

**Background:**

Liver cirrhosis is a leading cause of morbidity, premature mortality and acute care utilization in patients with digestive disease. In the province of Alberta, hospital readmission rates for patients with cirrhosis are estimated at 44% at 90 days. For hospitalized patients, multiple care gaps exist, the most notable stemming from i) the lack of a structured approach to best practice care for cirrhosis complications, ii) the lack of a structured approach to broader health needs and iii) suboptimal preparation for transition of care into the community. Cirrhosis Care Alberta (CCAB) is a 4-year multi-component pragmatic trial which aims to address these gaps. The proposed intervention is initiated at the time of hospitalization through implementation of a clinical information system embedded electronic order set for delivering evidence-based best practices under real-world conditions. The overarching objective of the CCAB trial is to demonstrate effectiveness and implementation feasibility for use of the order set in routine patient care within eight hospital sites in Alberta.

**Methods:**

A mixed methods hybrid type I effectiveness-implementation design will be used to evaluate the effectiveness of the order set intervention. The primary outcome is a reduction in 90-day cumulative length of stay. Implementation outcomes such as reach, adoption, fidelity and maintenance will also be evaluated alongside other patient and service outcomes such as readmission rates, quality of care and cost-effectiveness. This theory-based trial will be guided by Normalization Process Theory, Consolidated Framework on Implementation Research (CFIR) and the Reach-Effectiveness-Adoption-Implementation-Maintenance (RE-AIM) Framework.

**Discussion:**

The CCAB project is unique in its breadth, both in the comprehensiveness of the multi-component order set and also for the breadth of its roll-out. Lessons learned will ultimately inform the feasibility and effectiveness of this approach in “real-world” conditions as well as adoption and adaptation of these best practices within the rest of Alberta, other provinces in Canada, and beyond.

**Trial registration:**

ClinicalTrials.gov: NCT04149223, November 4, 2019.

## Background

Liver cirrhosis is a chronic condition that results from vascular and hepatocellular injury, and leads to progressive hepatic fibrosis. It is a major cause of morbidity and premature mortality in patients with digestive disease [1, 2]. Caused by a range of potential insults including alcohol, hepatitis B and C, and non-alcoholic fatty liver disease, cirrhosis is characterized by liver related complications from portal hypertension and hepatic insufficiency (i.e. ascites, variceal bleeding, hepatic encephalopathy, infections) [3, 4]. Many patients also have broader health needs that complicate their presentation including frailty, addictions, psychosocial challenges, and financial and employment instability [5–7]. Not surprisingly, this progressive, largely incurable disease results in an exceedingly poor quality of life for patients and their caregivers [8, 9] as low as that reported for patients with advanced metastatic cancer [10].

A number of recent studies have brought attention to the high rates of acute care and resource utilization associated with cirrhosis [5, 11–15]. In a recent prospective cohort study of 14 centers across North America, 90-day readmission rates were reported at 53% [14]. This data parallels provincial Alberta Health Services (AHS) administrative data for a 1-year period (2015–2016) which revealed readmission rates of 44% at 90 days [14] (Carbonneau M, Davyduke T, Tandon P, Ma M, Den Heyer V, Newnham K, et al: Impact of Specialized Multidisciplinary Care on Cirrhosis Outcomes and Acute Care Utilization, unpublished). AHS data analysis over that time period reported a mean hospital length of stay of 13.7 days and an annual inpatient cost of approximately $120 million [16]. Notably, the costs for cirrhosis were comparable to the $131 million in costs for chronic obstructive pulmonary disease in the same year, a condition already identified as a priority area for many healthcare organizations including AHS [17]. At $28,205, the mean cost per admission for Albertans with cirrhosis was 3.5 times the cost of a standard hospital stay.

In response to this “crisis in the making” [18], there has been much attention focused on strategies to reduce care gaps, improve quality of care and reduce readmissions in patients with cirrhosis [19–21]. For inpatients, these gaps can be summarized into three priority areas: i) management of cirrhosis complications, ii) management of broader health needs such as frailty, alcohol use disorder (AUD) and advance care planning and iii) preparation for transition of care into the community, including structured education and timely follow-up post discharge. There has been extensive data to support the presence of these care gaps in cirrhosis and emerging data to support the positive impact of solutions in each of these areas. For example, despite robust evidence that guideline-based care improves patient outcomes, including for those patients with cirrhosis, evidence-based guidelines are only utilized in 30–60% of patients [22–25]. An example of a successful solution in this area has been an electronic order set intervention implemented by Tapper et al. focused on inpatient management of hepatic encephalopathy and prophylaxis against spontaneous bacterial peritonitis. This intervention reduced 30-day readmission rates by 40% [26]. With regards to gaps in broader health needs, although greater than 50% of cirrhosis-related admissions can be attributed to alcohol [13], and it is clear that abstinence reduces hepatic decompensation, readmissions and mortality, in two recent series < 15% of patients received treatment for AUD [27, 28]. Similarly, malnutrition, sarcopenia and frailty are strong predictors for morbidity and mortality in patients with cirrhosis [29]. The management of malnutrition has been associated with lower hospital readmissions but structured care in this area is infrequent [30–32]. Lastly, there are gaps in in preparing patients for transition of care into the community. While it is clear that education improves patient knowledge, ability to self-manage their disease, and can even reduce the risk of re-hospitalization in populations such as heart failure, no hospital site in Alberta offers standardized discharge teaching to patients with cirrhosis [33, 34]. Moreover, the minority of patients have follow-up appointments arranged with primary and/or specialty care prior to discharge despite evidence that timely follow-up reduces mortality [35].

Standardized order sets represent a potential vehicle for care optimization. Clinical order sets are pre-defined templates that standardize and expedite the ordering process, guiding clinicians to facilitate guideline-based care [36, 37]. The use of standardized order sets, particularly those incorporated into an electronic clinical information system (CIS), have been associated with a reduction in in-hospital mortality, improvement in the delivery of care, and increased adherence to clinical practice guidelines [38–42]. While there have been several studies examining the impact of standardized order sets on the management of individual cirrhosis complications including variceal hemorrhage, hepatic encephalopathy and spontaneous bacterial peritonitis, to date we are unaware of a study that has covered a comprehensive list of cirrhosis complications and extended beyond these complications to address selected broader health needs and optimization of the transition of care into the community [43–45].

## Aims and objectives

**Fig. 1:**
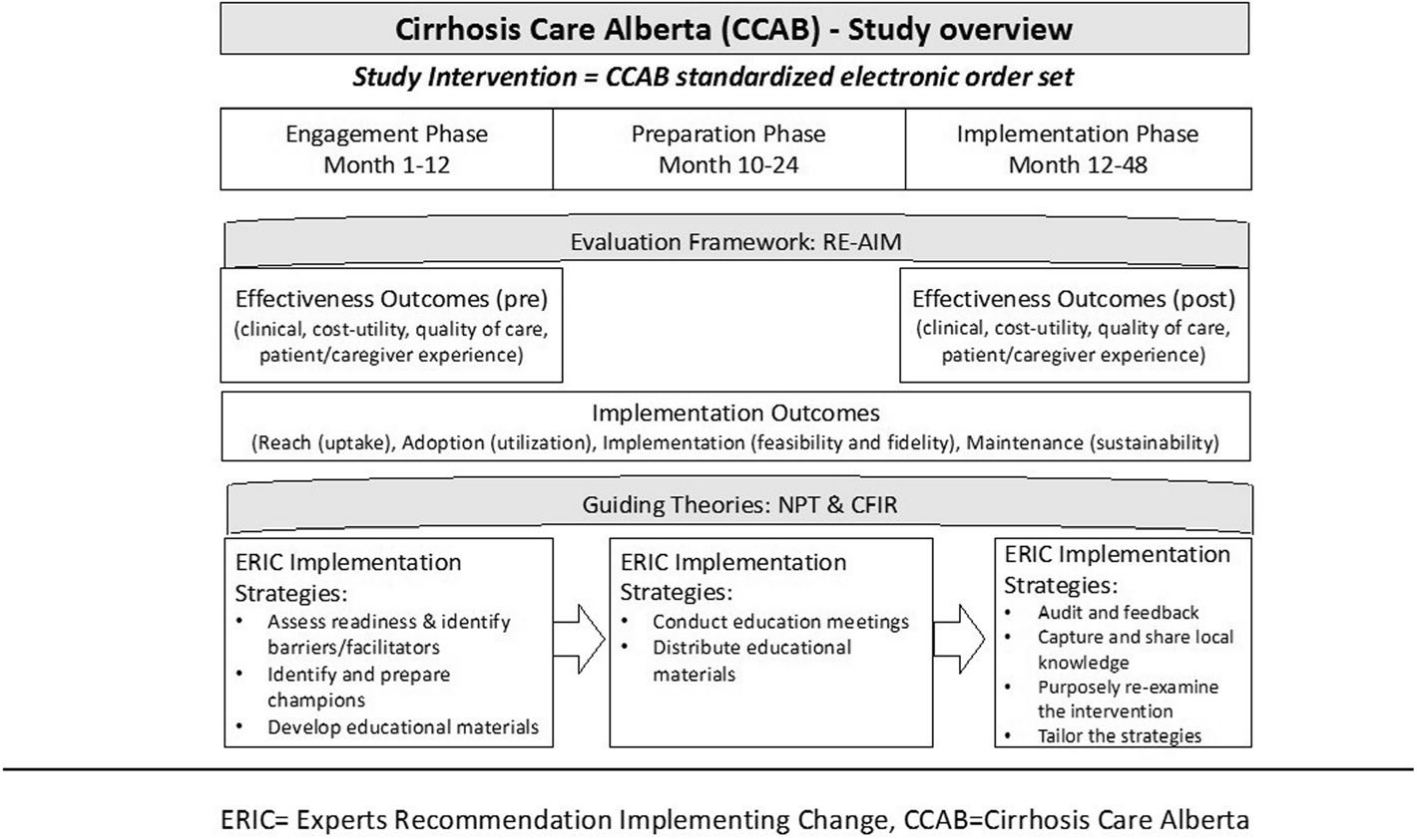
Cirrhosis Care Alberta Study Overview

The primary aim of this study is to evaluate the effectiveness of the Cirrhosis Care Alberta (CCAB) order set intervention in reducing 90-day cumulative length of stay (LOS). As this is a hybrid study, we will also assess the contextual factors that affect implementation in a real-world clinical setting (Fig. 1). Our research questions address the effectiveness of the intervention in addition to implementation processes and outcomes.

### Effectiveness questions

As compared to usual care, will a CIS facilitated rollout of the CCAB order set:

1. … reduce the 90-day cumulative hospital length of stay for patients with cirrhosis?
2. … reduce the occurrence of other clinical outcomes including hospital admission rate, median length of stay, readmission rate at 30 and 90 days, time to readmission, emergency department (ED) visit rate, outpatient visit rate and mortality?
3. … increase the quality of care (i.e. proportion of pre-defined process and clinical outcome-based quality measures that are fulfilled)?
4. … improve the patient/caregiver experience with care?
5. … demonstrate cost-utility?

### Implementation questions

1. … have uptake/reach among eligible individuals at each hospital site?
2. What contextual factors will influence adoption, reach, implementation fidelity, implementation feasibility and maintenance of the CCAB order set rollout?

## Methods/design

### Study design

**Fig. 2:**
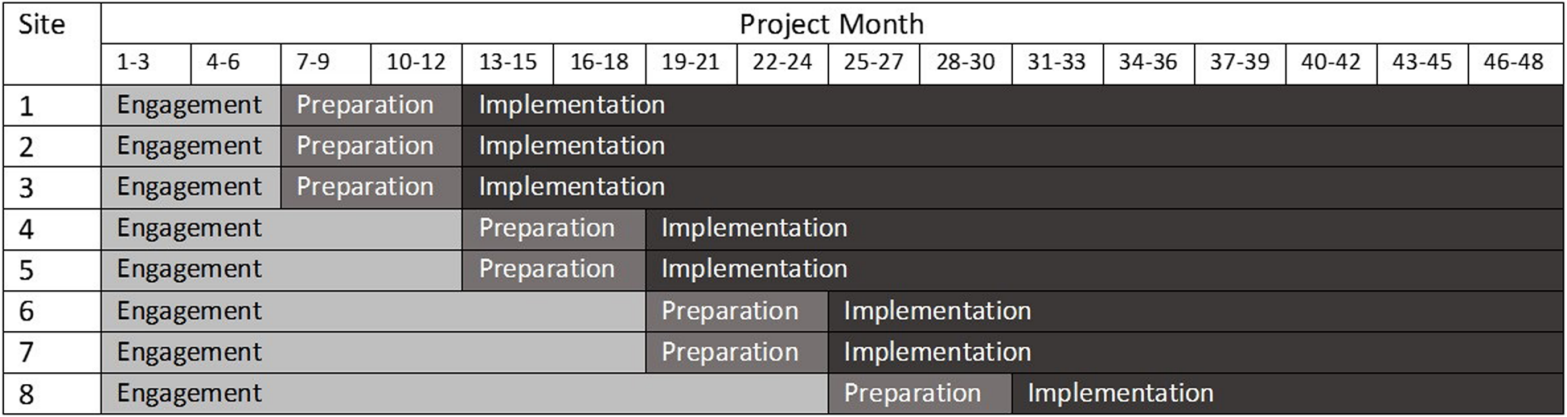
Project Phase Timeline

This 4-year pragmatic non-randomized study is guided by a hybrid type I effectiveness-implementation design. This study design evaluates the effects of the Cirrhosis Care Alberta (CCAB) order set intervention on relevant outcomes, while at the same time observing and collecting data on the implementation [46–48]. Pragmatic across multiple aspects of the Pragmatic-Explanatory Continuum Indicator Summary 2 (PRECIS-2) tool kit [49], and guided by various checklists from the EQUATOR network [50–52] (see Additional files 1, 2, 3), the intervention has been designed to be incorporated into clinical practice in a sustainable manner using existing infrastructure and the leadership of site champions (healthcare providers caring for patients with cirrhosis and site administrators). In view of contextual variations and other practical constraints, most notably the coincidental roll out of a unifying Epic® based CIS platform across the province, a staged implementation was chosen (Fig. 2) with three phases including engagement, preparation and implementation. These stages are further described in the “description of study phases and implementation strategies” section and in Fig. 1. The staged implementation and analysis allows comparison of the impact of the phases within and between study sites.

### Guiding theoretical frameworks

The overarching evaluation of the order set implementation is guided by the Reach-Effectiveness-Adoption-Implementation-Maintenance framework (RE-AIM) [53] summarized in Table 2. The Normalization Process Theory (NPT) and Consolidated Framework for Implementation Research (CFIR) will be used to understand the implementation process in greater depth. NPT is a theory of implementation that aims to explain the behaviour of individuals and groups around embedding and sustaining a new practice, with specific focus given to explaining how the practice impacts relationships within the clinical setting, how it is integrated into normal workflow, and how it is understood by implementers [54]. CFIR is a conceptual framework that provides a structured means of facilitating the design, implementation and evaluation of interventions across five domains: (i) intervention characteristics, (ii) outer setting, (iii) inner setting, (iv) characteristics of individuals, and (v) process of implementation [55]. In conjunction with NPT, we will use CFIR to understand both individual and organizational contextual factors that influence implementation of the CCAB order set.

NPT allows exploration of the generative mechanisms (coherence, cognitive participation, collective action, reflexive monitoring), examples of self-organizing mechanisms in complex adaptive systems. It also incorporates an understanding of differences in context and how implementation variably happens in different settings, and over time [56].

### Study setting

Health care in the province of Alberta is publicly funded and includes access to medically necessary services for all residents. Alberta Health Services (AHS), is a single health authority responsible for decision-making and delivery of healthcare services at hundreds of sites in the province (hospitals, as well as various continuing care and community health programs). It is organized into five geographic zones (North, Edmonton, Central, Calgary and South) [57]. In late 2019, AHS introduced a province-wide Epic® based CIS known as Connect Care to consolidate 1300 clinical information systems in use across the province. The implementation of Connect Care will facilitate consistent practices and access to patient medical records across hospital care sites in the province, and will be implemented in nine waves across all five provincial health zones from November 2019 until the Fall of 2022.

Eight hospital sites in Alberta have been selected to participate in the study. Implementation of the CCAB order set intervention at the sites will be facilitated by the Connect Care rollout. The selection of hospital sites has been guided by a consideration of (i) urban vs. rural communities, (ii) type of hospital (academic vs. community), and (iii) prevalence of cirrhosis. The selection has also considered the importance of having representation from the five health zones comprising AHS. Table 1 shows the selected hospitals in each zone as well as the 2017 administrative data estimates of the prevalence of cirrhosis in each zone.

**Table 1:** CCAB Project Hospital Sites

Zone	Age-Standardized Cirrhosis prevalence n(%)	Implementation sites
Edmonton Zone	3271(0.27)	4 (2 academic and 2 community hospitals)
Central Zone	1115 (0.24)	1 Community hospital
North Zone	986 (0.25)	1 Community hospital
Calgary Zone	3780 (0.26)	1 Academic hospital
South Zone	775 (0.27)	1 Community hospital

### Participant eligibility criteria

Adult patients (≥ 18 yrs.) admitted to a hospital site between January 2019 and January 2023, with a diagnosis of cirrhosis as determined by a validated ICD-10 based coding algorithm [58–61] will be included in the study cohort. After review by our local research ethics board, the need for direct patient consent has been waived for the development of the main study cohort.

Informed consent will be required for patients who participate in surveys, focus groups or interviews. Patient eligibility for these parts of the study will require a history of hospitalization during the study period with a diagnosis of cirrhosis based on compatible clinical presentation, FibroScan® and/or liver biopsy. Informed consent will also be required for healthcare providers (including physicians, nurses and other allied health) who participate in surveys, focus groups or interviews. Consenting healthcare providers will be eligible to participate if they are employed in Alberta and provide care to patients with cirrhosis.

### Intervention overview – the CCAB order set

**Fig. 3:**
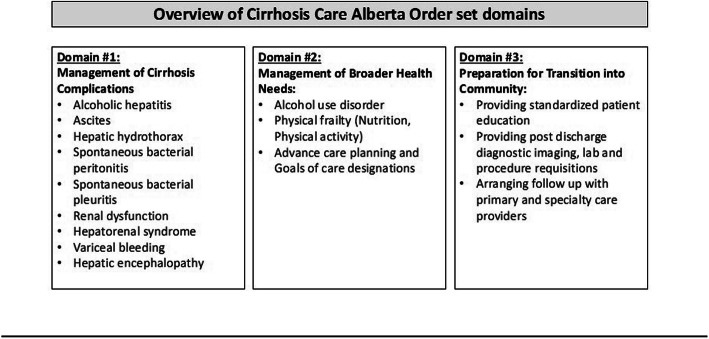
Overview of Cirrhosis Care Alberta (CCAB) Order Set Domains

The CCAB intervention, a standardized CIS order set, was developed through an iterative process in collaboration with a team of over 100 stakeholders in Alberta, including clinical experts such as physicians, other health providers, and nursing staff, as well as operational leadership, and patient advisors. The order set, intended for use during hospitalization of any patient with cirrhosis, includes three core domains (Fig. 3) – i) cirrhosis complications including ascites, hepatic encephalopathy and variceal bleeding, ii) broader health needs such as frailty, alcohol use disorder management, and advance care planning and iii) preparation for transition of care into the community including orders for the provision of standardized education and booking timely follow-up prior to discharge.

### Description of Study Phases & Implementation Strategies

The Expert Recommendations for Implementing Change (ERIC) includes a consensus-derived compilation of implementation strategies [47]. Selected ERIC strategies will be utilized to support implementation of the CCAB order set into routine clinical practice throughout three distinct study phases: (i) engagement, (ii) preparation, and (iii) implementation.

#### Phase 1 - engagement

Phase 1 will take place from months 1 to 24, prior to the CCAB order set integration within the CIS at each site (see Fig. 2). During this engagement phase, baseline data will be collected, and readiness assessments will be performed at each of the eight study sites.

*ERIC Strategies within this phase: Assess for readiness and identify barriers and facilitators, Identify and prepare champions, Develop educational materials*

During planned site visits, readiness assessments will be carried out using qualitative methods including individual interviews and focus groups (guided by the constructs of NPT and CIFR), with the aim of understanding individual site context and identifying barriers and facilitators to implementation of the order set. Site visits will also be used to identify current practices in each of the three order set domains (cirrhosis complications, broader health needs, preparation for transition of care into the community). Site champions will be identified. These champions will come forward to support the work and facilitate implementation and ongoing communication between the project team and front-line staff. Finally, educational tools such as webinars and handouts focused on the three domains of the order set will be developed in collaboration with site champions and subject matter experts. Co-development of these tools will facilitate ongoing engagement at the sites and will support the implementation.

#### Phase 2 - preparation

The preparation phase of the study will commence within 6 months prior to the planned electronic order set implementation at each site **(**Fig. 2).

*ERIC Strategies within this phase: Conduct education meetings with health care providers. Distribute educational materials.*

Using the tools developed in Phase 1, the study team will facilitate pre-implementation education that considers the preferred timing and learning modalities of each study site, as well as the specific learning needs of individuals (e.g., physicians and nurses). Education will include review of the order set and its components. For example, education meetings for prescribers will involve teaching around AUD diagnosis and use of pharmacologic therapies to reduce relapse, while nurses will receive education about screening and having conversations with patients about AUD. At the request of our study sites, educational materials will be distributed in a variety of formats (videos, webinars, handouts) and will be hosted on easily accessible web-based platforms.

#### Phase 3 – implementation

The final phase of the study will commence with “go-live” of the CCAB order set in the provincial CIS at each site. The order set will be implemented using a staged approach, timing determined by timing of CIS implementation across the province (months 12–33). Outcome effectiveness measures will be collected during this phase.

*ERIC Strategies within this phase: Audit and provide feedback. Capture and share local knowledge. Purposely re-examine the implementation. Tailor strategies.*

As sites enter phase 3, site champions will be invited to participate in monthly learning sessions (conducted virtually with representatives from each site) to discuss their experience, including barriers, facilitators, and lessons learned. Collection of Key Performance Indicators (KPIs) will occur quarterly and be shared with sites on electronic scorecards representing audit and feedback. The scorecards will include a combination of standard KPIs (e.g. order set utilization, length of stay) and site prioritized KPIs (e.g. performing a screen for alcohol use disorder). The rationale for including site prioritized KPIs is to promote site engagement and ownership of practice change. In the learning session following the quarterly release of scorecards, the teams will share learnings with each other and develop action plans to tailor implementation strategies at their sites. These Plan-Do-Study-Act (PDSA) cycles will continue quarterly throughout the implementation phase.

### Evaluation

This hybrid trial evaluation is guided by the RE-AIM framework as summarized in Table 2. The outcome measures and data analyses plans are classified into effectiveness and implementation outcomes in accordance with the research questions.

**Table 2:** Overview of the Cirrhosis Care Alberta (CCAB) evaluation methods

RE-AIM Domains	Outcomes	Measures	Data sources/ tools	Analytic Methods	Study Phase		
					1	2	3
Reach	Uptake	Proportion of care providers who participate in education interventions for the order set,	Program data, Administrative data,	Descriptive statistics		X	X
		Proportion of cirrhosis patients being managed using the CCAB order set	Chart reviews				
Effectiveness	Clinical and Cost-Utility	I.e.. Cumulative hospital LOS and readmission rates per patient year,	Administrative data,	Interrupted time series,	X		X
			Chart reviews	Incremental Cost-Effectiveness Ratio			
		Cost of care (health system perspective) QALYs gained					
	Patient reported outcome measures (PROMs)	Health-related quality of life,	Post-discharge phone surveys, EQ-5D and CTM questionnaires,	Descriptive and inferential statistics,	X		X
		Patient/family and provider experience					
				Framework and thematic analyses			
			Qualitative interviews				
	Quality Measures	Quality of care (eg. appropriate prescribing of rifaximin, screening for frailty, time to post discharge follow up)	Chart reviews	Interrupted time series	X		X
Adoption	Utilization	Number of hospital sites that use the orders sets/Number of hospital sites enrolled,	Administrative data,	Descriptive statistics,			X
			Qualitative interviews	Framework and thematic analyses guided by NPT and CFIR			
		Processes and determinants of adoption					
Implementation	Feasibility	Processes and determinants of feasibility and acceptability from patients, caregivers and health care providers’ perspectives	Qualitative interviews	Framework and thematic analyses guided by NPT and CFIR	X	X	X
	Fidelity	Proportion of order sets prescribed that adhere to the three core-components of the standardized CCAB order set	Administrative data	Descriptive statistics			X
Maintenance	Sustainability	Proportion of cirrhosis patients being managed using the CCAB order set 6 months after the research team has completed supported rollout	CCAB program implementation data,	Descriptive statistics,			X
				Framework and thematic analyses guided by NPT and CFIR			
			Qualitative interviews				

*CCAB* Cirrhosis Care Alberta, *CFIR* Consolidated Framework for Implementation Research, *EQ-5D* EuroQol- 5 Dimension, *NPT* Normalization Process Theory, Study Phases: 1 = Engagement, 2 = Preparation, 3 = Implementation

### Outcome measures

1. **Effectiveness outcomes:**
  - **Primary:** The cumulative 90-day hospital length of stay (LOS)
  - **Secondary:**
    i. Clinical outcomes including hospital admission rate, median length of stay, readmission rate at 30 and 90 days, time to readmission, emergency department (ED) visit rate, outpatient visit rate and mortality
    ii. Health care utilization and cost utility
    iii. Quality of care as evaluated by quality measures from the three core order set domains
    iv. Patient and caregiver experience with the intervention
2. **Implementation outcomes:**
  - Reach (Uptake)
    i. The proportion of cirrhosis patients being managed using the CCAB order set, measured as the number of order sets used/ the number of patients eligible for orders set use
    ii. The proportion of care providers who participate in education interventions for the order set, measured as the number of care providers who participate in education interventions for the order set / number invited
  - Adoption (Utilization):
    i. The proportion of hospital sites that use the order sets measured as the number of hospital sites that use the order sets/number of hospital sites enrolled
    ii. Qualitative evaluation of processes and determinants of adoption guided by the NPT and CFIR
  - Implementation:
    i. Feasibility/ acceptability: Qualitative evaluation of processes and determinants of feasibility and acceptability of order set implementation from the perspectives of patients, caregivers and health care providers’ guided by the NPT and CFIR.
    ii. Fidelity: Proportion of prescribed orders that adhere to the three core domains of the standardized CCAB order set, measured as the number of order sets that use orders in each of the three core domains/number of patients eligible for order set use
  - Maintenance (Sustainability): Proportion of cirrhosis patients being managed using the CCAB order set 6 months after the research team has completed supported rollout (measurement similar to “reach”)

### Sample size, data collection and analysis

#### Effectiveness outcomes - clinical outcomes, cost-utility

i.) **Sample size**

Consistent with the type 1 hybrid trial design, the overarching sample size calculation is based on the cumulative 90-day hospital length of stay (LOS) [48]. Based on 2015/2016 AHS administrative data (4176 cirrhosis-attributed admissions for 2652 patients at study sites) [62] and CCAB implementation time per site, we estimate a pre-intervention cohort of 3975 patients and post-intervention cohort of 3975 patients (n = 7950). Our calculations tested our ability to detect a 3-day reduction in average 90-day cumulative LOS from our historically observed average of 13.7 days. Computer simulations re-sampled historic LOS data to assess power to detect proposed effects for plausible scenarios using interrupted time series models with random intercepts for sites and an over-dispersed Poisson distribution. A scenario that assumed that 50% of eligible patients would receive the pathway (our most conservative estimate) and that there would be a rapid and sustained uptake of the pathway in the two years following implementation had > 90% power to detect an effect in the three largest sites and the whole cohort [63].

ii.) **Data collection and analysis**

The index population will be identified at admission with a validated Canadian algorithm of ICD9 and ICD10 codes (overall accuracy 87%) [58]. We will analyze administrative data (n = 7950) using linear effect modeling that accounts for patient factors and observation clustering within sites [64]. We will use segmented regression analyses of interrupted time series to model 90-day cumulative LOS and hospitalization rate in each period [65], comparing post-intervention changes with pre-intervention secular trends [66]. We will evaluate and account for autocorrelation or other serial dependencies in data. Each of five geographic zones and eight hospital sites will serve as its own control, enabling us to identify effectiveness within each jurisdiction. We will also combine site series into generalized linear mixed effects models [64], with a random intercept for each site, fixed effects for time, and an indicator variable for pre-and post-intervention periods for each site.

Cost-utility evaluation will estimate expected incremental cost per Quality Adjusted Life Year (QALY) gained by implementing CCAB, within the Net Benefit Regression framework [67]. Analysis will control for differences in relevant patient cohort characteristics. The evaluation will adopt a health system perspective and within-study analysis (only study cohort costs and outcomes), comparing resource use and health outcomes (health-related quality of life, mortality) for CCAB and usual care cohorts. These will be combined to calculate within-study expected QALY for each cohort. Methods for economic evaluation (e.g., discount rate choice, uncertainty characterization, results presentation) will adhere to recent reference case recommendations (Canadian Agency for Drugs & Technologies in Healthcare) [68]. Stochastic analysis will be implemented with non-parametric bootstraps. Results will be presented as Expected Net Health Benefit and Cost Effectiveness Acceptability Curve. We will also report Value of Information to characterize residual decision uncertainty on value to Alberta of CCAB spread and scale at study completion.

#### Effectiveness outcome - quality of care measures

i.) **Sample size**

We will randomly sample at least 50 charts per site. This will provide 95% confidence intervals with widths no greater than +/− 10% for percentages of patients meeting quality measures (QMs) within each site.

ii.) **Data collection and analysis**

Improvements in quality of care will be evaluated using QMs for each of the three CCAB order set domains [69] (see Table 3)**.** The majority of QMs will be obtained from administrative and chart review data audits from randomly selected charts and will take place quarterly during the study. The data from these audits will be used to promote change at each site. The central data collection team will record survey results in a secure AHS database for analysis. We will use inverse sampling weights to estimate the percent of patients attaining the QMs for the province. Administrative data will be analyzed for clinical outcomes, with additional analyses to compare change in QMs based on patient-reported outcomes, using linear mixed effects models that account for patient factors and observation clustering within sites [64].

**Table 3:** Sample of Quality Measures (QMs)

Cirrhosis Care Alberta Order Set Domain	Quality Measure Definition
**Management of Cirrhosis complications**	
Ascites	*Patients undergoing large volume paracentesis (> 5 l removed) should receive intravenous albumin (6–8 g per liter removed)
Hepatic hydrothorax	*Patients with ascites and/or hepatic hydrothorax should be managed with both sodium restriction and diuretics (unless there is a contraindication for diuretics)
Spontaneous bacterial peritonitis	*Hospitalized patients with ascites, with an ascitic fluid polymorphonuclear count of ≥250 cells/mm^3^, should receive empiric antibiotics and albumin within 12 h of the test result. The first dose of albumin should be 1.5 g per kg body weight followed by a second infusion of 1.0 g/kg on day 3
Spontaneous bacterial pleuritis	*Hospitalized patients with a pleural fluid polymorphonuclear count of ≥500 cells/mm3 (or ≥ 250 cells/mm3 with positive culture), should receive empiric antibiotics within 12 h of the test result
Renal dysfunction	Patients with acute kidney injury should be given an intravenous albumin challenge of up to 100 g × 2 days.
Hepatorenal syndrome	Patients with cirrhosis and hepatorenal syndrome who have a MAP of < 65 mmHg should receive a combination of vasoconstrictors and albumin therapy
Variceal bleed	*Patients with cirrhosis who survive an episode of acute variceal hemorrhage should receive a combination of EVL (endoscopic variceal ligation) and β -blockers
Hepatic encephalopathy	*Patients who are discharged after an acute episode of hepatic encephalopathy should receive secondary prophylaxis with lactulose and/or rifaximin
Alcoholic hepatitis	Patients with ETOH hepatitis and a MELD score of > 20 should be considered for prednisone therapy provided there are no contraindications
**Management of Broader health needs**	
Advance care planning and goals of care	Patients admitted with cirrhosis should have goals of care documented
Alcohol use disorder	*Patients with cirrhosis should receive counseling or be referred to a substance abuse treatment program within 2 months of positive screening
Nutrition and physical activity optimization	Patients admitted with cirrhosis should be prescribed a high protein/high calorie (± as needed, a low sodium) diet
**Preparation for transition into the community**	
Standardized cirrhosis education for patients/caregivers	Patients with cirrhosis should receive cirrhosis education prior to discharge
Post-discharge laboratory, diagnostic imaging and endoscopy appointments	Patients with cirrhosis should receive information about when to have lab work done post discharge
Post-discharge follow-up with primary and/or specialty care	*Recently discharged patients with cirrhosis should have a clinic visit with a health care provider within 4 weeks of discharge

Table includes a sample of the Quality Measures (QM) that will be evaluated from each domain of the Cirrhosis Care Alberta (CCAB) order set. Additional QMs will also be evaluated. QMs were selected based on consensus by either: *Practice Metrics Committee of the American Association for the Study of Liver Diseases [70], or consensus between the CCAB study team members

#### Effectiveness outcome - patient and caregiver experience with the intervention

i.) **Sample size**

Purposeful maximum variation sampling will be used to ensure recruitment of a diverse group of patients and caregivers (rural, urban, socioeconomic status) to explore their experiences with the intervention. We estimate needing 40–50 participants to achieve saturation, typical for this methodological approach and appropriate given the variation in cirrhosis disease experiences [70]. This data will be supplemented by patient phone surveys done in the pre and post implementation periods where patients with cirrhosis admitted to study sites will be contacted 7–14 days post-discharge and administered a health-related quality of life measure (EQ-5D) [71] and care transitions survey (CTM-15) [72] to assess their experience with care. For patient phone surveys, we will take a convenience sample of patients pre and post implementation of the intervention based on the capacity of units to identify patients.

ii.) **Data collection and analysis**

The data collection will begin after 1–2 months post-implementation at each study site (to allow for intervention uptake). Patients and caregivers will be invited to participate in individual semi-structured qualitative interviews focusing on key areas such as self-management, self-efficacy for cirrhosis, and relationships with healthcare providers using qualitative description methods [70, 73, 74]. For comparable historical context, only patients with at least one hospital admission prior to implementation of the order set will be invited to participate. For participant convenience, interviews will be in-person or virtual, with these interviews being recorded and transcribed verbatim. Participant data will be coded into meaningful segments, then organized into areas of similar patterns or themes [75]. We will examine areas of commonality and difference in thematic analysis based on factors such as demographics, socioeconomic status and cirrhosis/hospitalization experiences [76, 77]. Data collection and analysis will be concurrent and iterative to enable refinement of the recruitment process and semi-structured interview guide [77].

#### Implementation outcomes

i.) **Sample size**

Over the course of the project we anticipate surveying approximately 280 service providers (physicians, nurses and other health professionals), estimates derived from the number of providers involved with the management of cirrhosis at the sites. The qualitative evaluation of implementation will use a similar sampling strategy with the evaluation of patients’ experience described previously.

ii.) **Data collection and analyses**

Implementation data will be collected from a variety of sources including AHS administrative database, chart reviews, provider surveys using validated questionnaires and interviews with physicians and nurses. The CCAB project repository and participant observations from throughout the project will be leveraged to obtain data on implementation context and processes. Implementation data will also be collected during monthly virtual learning session meetings and any on-site visits that occur during the study phases. The quantitative data will be analyzed descriptively to monitor reach, adoption and implementation fidelity following the PDSA cycles. Qualitative data will be analyzed using a framework analytic approach to evaluate the contextual factors impacting adoption, reach, implementation fidelity, implementation feasibility and maintenance of the CCAB order set rollout [78].

## Discussion

Across a range of populations, there is evidence to support that order set delivery via an electronic medical record reduces medical errors, improves adherence to clinical practice guidelines, and has a positive impact on patient outcomes including a reduction in patient mortality [40, 79–81]. There has also been promising data to support the impact of order set delivery in patients with cirrhosis. To date however, the majority of cirrhosis studies have focused on more of an explanatory research design, filling the gaps in only one or two cirrhosis associated complications at time [26, 43–45]. The current pragmatic study builds off of the excellent work that has already been done, adding several novel aspects in the implementation of a comprehensive and pragmatic best practice intervention for hospitalized patients with cirrhosis.

The CCAB order set is unique in that it includes a comprehensive set of best practice orders. In addition to including orders for nine of the top cirrhosis complications that patients are admitted for, uniquely, it also contains orders focused on broader health needs including enhanced diet and activity orders as well as a specific order panel for the management of alcohol use disorder. Moreover, it includes orders to optimize care coordination and transition of patients into the community with elements such timely notification of primary care providers and patient instructions for follow up appointments and home self-management. Additionally, the order set is supported by educational materials such as written and video materials developed for patients and providers with a goal of facilitating discharge education. It is anticipated that these educational materials will improve knowledge not only for patients, but also for nurses and will be valuable tools for additional scale and spread of the intervention once the current study is complete.

The scope of the study is broad, including all adult patients with cirrhosis admitted at eight hospital sites, with representation from each of the five zones across the province. Universal access to healthcare services within Alberta will reduce patient selection bias. Importantly, the study spans urban as well as suburban sites, allowing us to evaluate discrepancies in the patient populations, quality indicators and clinical outcomes that may be present across these sites. The CIS will be the vehicle for the order set intervention and the study timing is therefore aligned with the CIS roll-out. We anticipate that the qualitative interviews that will occur during the study period will provide a wealth of information about the acceptability and barriers that come up during such a large scale CIS roll-out. This will be potentially useful to other sites who are transitioning to a hospital CIS.

Another unique aspect from existing work is that the CCAB study uses a type I hybrid effectiveness-implementation design. A broad range of effectiveness outcomes will be evaluated including clinical outcomes, quality of care, health economic outcomes, health-related quality of life, and experience with the intervention. By using multi-methods quantitative and qualitative evaluation, the hybrid design allows for a concurrent evaluation of implementation strategies and contextual factors that impact the effectiveness of the intervention. Site champions will be crucial in supporting implementation and sustainability of the intervention into routine clinical practice. Diverse implementation strategies will be used to keep site champions and local teams engaged, and information about the success of these strategies will be captured using the hybrid study design. The multi-methods evaluation allows for patient and provider input to be prioritized throughout the project. The participatory, patient-focused approach to continuous evaluation and improvement of implementation strategies will ensure that the order set is optimally implemented to the benefit and satisfaction of patients.

As the study aims to implement in a complex real-world situation as opposed to a controlled setting, the Pragmatic-Explanatory Continuum Indicator Summary 2 (PRECIS-2) tool kit [49] has been applied to align the design towards a more pragmatic than an explanatory trial (Additional file 1). As with other pragmatic trials, several limitations to the study design exist, including the lack of randomization. The rollout of the CIS has been fixed by AHS and has by necessity determined when each site begins the intervention. Significant competing priorities within the organization such as the COVID-19 response will inevitably impact the implementation timelines. Moreover, although we are recording quality measures across all three domains of the order set, given the complexity of the intervention and implementation context, our ability to attribute causality to any one component of the order set will be limited.

In conclusion, the findings from this unique and ambitious project are expected to contribute to existing knowledge on the effectiveness and feasibility of implementation of best practices in cirrhosis care. It is anticipated that technical and allocative efficiency of care will be positively impacted, even within the 4 year study duration [82]. Lessons learned and materials developed during the rollout of the CCAB intervention will serve as a framework for potential future spread of the intervention. With an aim to improve equitable access and enhance allocative efficiency of health care expenditure across the province of Alberta, we anticipate that the CCAB order set, supporting materials and implementation processes will represent an important and innovative step forward in closing the gaps in cirrhosis care not only in our province but also in various other contexts across Canada and the world [82, 83].

## Supplementary information

**Additional file 1.** PRagmatic Explanatory Continuum Indicator Summary (PRECIS-2). PRECIS-2 is a tool to help trialists designing clinical trials consider where they would like their trial to be on the pragmatic/explanatory continuum

**Additional file 2.** The TIDieR (Template for Intervention Description and Replication) Checklist. TIDieR reports details of the intervention elements of the study

**Additional file 3.** Standard Protocol Items: Recommendations for Interventional Trials (SPIRIT) Checklist. SPIRIT reports on items to address in a clinical trial protocol

## Data Availability

The datasets analysed for the current study protocol are available from the corresponding author on reasonable request.

## Abbreviations

AHS: Alberta Health Services
CCAB: Cirrhosis Care Alberta
CIS: Clinical Information System
CFIR: Consolidated Framework for Implementation Research
EQ-5D: EuroQol- 5 Dimension
ERIC: Experts Recommendation Implementing Change
HNHC: High Need, High Cost
ICD-10: International Classification of Diseases (10th Revision)
KPI: Key performance Indicators
KT: Knowledge Translation
LOS: Length of Stay (on admission)
NPT: Normalization Process Theory
ORIC: Organizational Readiness to Implement Change
PDSA: Plan-Do-Study-Act
PRECIS-2: Pragmatic-Explanatory Continuum Indicator Summary 2
RE-AIM: Reach-Effectiveness-Adoption-Implementation-Maintenance
