# Supplementary material for: The cirrhosis care Alberta (CCAB) protocol: implementing an evidence-based best practice order set for the management of liver cirrhosis - a hybrid type I effectiveness-implementation trial
Source: BMC Health Serv Res. 2020 Jun 18;20:558. doi: 10.1186/s12913-020-05427-8 (PMC7301349; doi:10.1186/s12913-020-05427-8)
Supplement: Supplementary file 1 — Additional file 1. PRagmatic Explanatory Continuum Indicator Summary (PRECIS-2). PRECIS-2 is a tool to help trialists designing clinical trials consider where they would like their trial to be on the pragmatic/explanatory continuum [file 12913_2020_5427_MOESM1_ESM.docx]

Additional file 1: Pragmatic assessment of the CCAB design using the PRECIS-2 domains

| PRECIS-2 Domain | Description of pragmatism |
| --- | --- |
| Eligibility | This effectiveness-implementation hybrid trial with real-world patients and providers. Participants (providers) will only be excluded if they opt out. The intervention is being designed and implemented to become the new and enhanced standard of care for all patients with cirrhosis. |
| Recruitment | The intervention will be built into current workflow and whether a patient received the intervention or not will be evaluated with implementation outcomes (i.e. whether the care providers prescribe and use the care bundle and whether the patients use the self-management tools that are provided). The existing infrastructure (emergency units, medical wards, human resources, etc.) will be used to administer the intervention to patients diagnosed with cirrhosis. |
| Setting | The eight study sites were selected to reflect the diversity of patients and organizational settings in the province (high and low volume centres, Covenant and AHS facilities, urban, inner city, transplant center, rural, etc.). The trial will not modify or control context but will evaluate implementation and effectiveness within the real-world context and complexities both at intervention and control (usual care) phases. |
| Organization | The trial will not modify organizational structures at the intervention or control phases. The intervention will be rolled out in phases to accommodate intrinsic differences including competing priorities that are bound to affect when each site will have the readiness to fully engage in the intervention. This pragmatic approach makes it possible to compare processes and outcomes within sites (before and after) as well as between sites despite these differences, given that all sites will become implementing sites at the final phase of the study. |
| Flexibility – Delivery | At all sites, the intervention will be delivered in such a way as to accommodate real-world contextual variations. For example, implementation at each site will be guided in part by Connect Care rollout. The Implementation of the intervention, including implementation strategies, will be dynamic to accommodate Connect Care as a facilitating contextual platform, rather than as a study contaminant (as would have been in a strictly controlled trial). However, these real-world contextual variations and nuances will be noted and accounted for during analysis and interpretation of findings. |
| Flexibility – Adherence | One of the aims of the intervention is to improve fidelity (adherence) with the standardized best practice care bundle using a participatory approach. No one will be excluded on the grounds of poor adherence, rather the provider participants, through strategies such as the learning collaborative meetings with audit and feedback, will co-develop pragmatic strategies to improve adherence. In order to ensure post-intervention sustainability, the collaborative meetings will not receive active influence from the study team beyond usual facilitation and initial guidance/training on the intervention. |
| Follow-Up | Given that the strategies include dynamic approaches such as using PDSAs which require active feedback from participants, the intensity of follow-up will be higher during the initial intervention phase than the usual care phase for each site. However, the staged roll out of the trial ensures that all participants receive same intensity towards the concluding phases of the trial. |
| Primary Outcome | The primary outcomes of the study are cumulative length of stay per patient year and hospital readmission rates. Reducing these outcomes is very relevant to both the physical and psychosocial well-being of patients, with potential to drastically improve quality of life. |
| Primary Analysis | The primary outcome measures will be evaluated using interrupted time-series analytic methods. All data collected and analyzed in this study is aimed at improving the length of stay per patient year and readmission rates of patients. Consequently, using the PDSA learning and improvement cycles, the data primarily serves the purpose of improving the quality of care, and secondarily will be used for evaluation of the intervention. The study design ensures that the improvement experienced in the course of the study is applied across all study sites (and participants) at the terminal phase of the project, as well as sustaining implementation of the best practice care bundle beyond the close of the project. |
